# The Importance of the Natural Feeding of Infants
*A Paper read at the (Biennial) Health Conference at the Royal Horticultural Hall, Westminster, June 1912.


**Published:** 1912-08-24

**Authors:** Harold Scurfield

**Affiliations:** Medical Officer of Health, Sheffield.


### THE IMPORTANCE OF THE NATURAL FEEDING OF INFANTS.*

By HAROLD SCURFIELD, M.D., Medical Officer of Health, Sheffield.

"Every mother is nurse to her own child unless either death or sickness be the let."—More's "Utopia."

All medical authorities agree that the proper food for an infant is its own mother's milk, and that the baby which is deprived of its natural sustenance is thereby exposed to real risk of early death or of permanently impaired health. The direful results of artificial feeding are well known to medical men. Let us get rid of the idea that the disastrous results of bottle-feeding are limited to the babies of the poor. It is not so, and I would be content to prove this from the experience solely of the artificially-fed babies of medical friends and acquaintances. My experience shows me that apathy and ignorance about breast-feeding and the want of perseverance to practice it extend to doctors' wives, and that the babies of medical men, in spite of presumably greater knowledge and care expended over the artificial feeding, suffer badly as a result of the system.

The risks run by the bottle-fed baby may be summarised as follows:—

A liability to all kinds of digestive disturbances, which may result in impaired digestion for life. A liability to rickets, with deformities lasting for life. A greatly increased liability to die during the first year of life from diarrhœa. A lack of vital resistance, causing it to succumb more easily to the various diseases which it may contract. Interference with the proper development of both the temporary and permanent teeth, with effects also lasting for life. Liability to scurvy; and liability to contract tuberculosis by the ingestion of tuberculous milk.

(N.B.—By Dr. Rotch's system raw milk is given, and the only means of obtaining tubercle-free cows' milk is from tuberculin-tested cows, a system which is not now practicable except on a very small scale.)

The foregoing list of well-recognised dangers is, no doubt, incomplete, but I think it is sufficiently formidable. What are we to say of the mother who deliberately refuses to feed her baby and condemns it to run these risks? She is certainly guilty of an offence under the Children Act for neglecting her child in a manner likely to be injurious to its health, although public opinion may not yet be ripe for prosecutions for failure to breast-feed. If the baby dies from some one of the ailments to which she has rendered it liable by her refusal, she is surely guilty of something akin to manslaughter.

If the mother does not know the risks to which she is exposing her child, is this ignorance the fault of the medical profession? If so, the sooner the remedy is found the better. There must be no uncertainty about the attitude of the medical profession in this matter. It must be clearly laid down that it is a crime for any woman, be she princess or peasant, who is able to do so to refuse to feed her own baby. It will then come to be recognised as a disgrace to the community if a woman is prevented from feeding her baby by having to go out to work. The most important point with which we have to deal is the alleged inability of women to feed their offspring. This inability is referred to in Playfair's "Midwifery" (edition 1881), and one could imagine it possible of the Early-Victorian mother. The inability is said to be increasing however, and to be due to the effects of "high civilisation." It is hard to believe that the woman of to-day, brought up with much more regard for physical development, is less able to nurse her babies than the Early-Victorian.

"The capacity for maternal nursing among the more cultured classes of women is steadily decreas-

* A Paper read at the (Biennial) Health Conference at the Royal Horticultural Hall, Westminster, June 1912.

ing," says Dr. Vincent. Holt, of New York, says the same. One wonders what is meant by civilisation and culture in this connection. Cannot a woman use her brain without getting atrophy of the mamma?

Everyone recognises the difficulty of distinguishing between inability and disinclination. The following extract from Dr. Vincent's book on "The Nutrition of the Infant," illustrates this point:—

"Finally, it should be remembered that, while maternal nursing is the ideal method, it is a mistake practically to force a really unwilling mother to undertake the suckling of her infant. Many young mothers, not unnaturally anxious to avoid the severe restrictions and disabilities nursing imposes, are unwilling to accept the duty. But when the importance of breast-nursing by the mother is properly put before them, they cheerfully accept the duties and bear with the inconvenience. On the other hand, it is seldom wise to urge the duty upon a mother when there is little prospect that she will properly discharge it."

This quotation is a good example of the half-hearted attitude of the medical profession in this matter. Fancy a doctor talking about a young mother being not "unnaturally" anxious to avoid nursing her children! Can anything be more unnatural?

There are many reasons why it is difficult to distinguish between inability and disinclination. First of all, we have to consider the dictates of fashion. The "woman who couldn't" has, perhaps, in the past been considered rather a superior sort of person, too intellectual for such an animal task. There has been much ignorance of the importance of breast-feeding, no doubt encouraged by the half-hearted attitude of the medical profession on the subject. The ignorance of fathers in this respect is quite an important factor. There has been shirking by women who did not wish to give up their usual pursuits, or to spoil their figures, or whose husbands did not wish them to be tied.

Secondly, there has been almost a conspiracy on the part of a section of the monthly nurses to discourage breast-feeding, partly to please fashionable mothers, partly to gratify a sort of perverted maternal instinct and to obtain complete control of the infant, partly because it is more comfortable for the nurse to heat up a bottle in her own room than to stand by the mother's bedside perhaps on a cold night during the 30 to 45 minutes which is often necessary to get the infant to take its first meals. Some monthly nurses go so far as to keep the baby from the breast for the first two days so as to avoid stimulating the secretion of milk.

The attitude of the monthly nurse is, no doubt, partly due to ignorance—a reflex of the ignorance and apathy of the medical profession on the subject. She does not know what the baby is losing, and does not hear of its subsequent history.

Thirdly, there has been, and is, great ignorance on the part of the medical profession in the practical details of infant management. The management of the infant is neither medicine, surgery, nor midwifery, and has received scant attention in the medical curriculum. It thus comes about that the management of the infant has been left to the monthly nurse, and if the latter has reported that "there seems to be no milk," or that "the milk is very thin," etc., the idea of breast-feeding is given up light-heartedly, and the doctor goes round saying, "How extraordinary it is that so many women can't feed their babies nowadays!"

The doctor, not being well up in the details, is not in a good position to fight a shirking mother or a tricky monthly nurse. The advent of women into the profession might have been expected to create an improvement, but an unmarried female doctor is not much better than a man in these matters, and when a woman doctor has had children of her own it has generally been when she is not in practice, and the value of her experience is therefore lost.

Another point is the ignorance of the mothers. At present the primipara is absolutely at the mercy of the monthly nurse. When fashion changes and the medical profession take more interest in the matter, it is to be hoped that a woman will come to her first confinement "armed and engined for the same" with all the necessary preparation.

Medical men who have given special attention to the matter find that a large proportion of the cases of alleged inability to breast-feed can be dealt with if the mother is in earnest. Thus difficulties due to depressed or sore nipples or supposed scanty supply can often be overcome by perseverance.

I wish in this connection to refer especially to the work of Professor Budin,* of Paris, who has shown that the supply usually meets the demand if the proper stimulus is applied to the breast.

This is where the difficulty of the medical man comes in. He cannot be always there to see if the nurse applies the proper stimulus by putting the baby to the breast at the proper intervals, and is more or less helpless when the nurse reports that there is no milk. Remember, this theory of the atrophy of the female breast is much more startling than anything that was brought out by the Committee on Physical Deterioration, and should not be accepted without very complete proof.

The first steps are to make breast-feeding fashionable, and to secure much more attention for this and other matters in connection with infant management in the medical schools. When this has been accomplished we shall be in a better position for judging as to the facts with regard to this alleged physical deterioration. For goodness' sake let us leave off talking of high civilisation and culture in this connection. Let us give it its proper title, whether it be physical deterioration or fast life. Women are supposed to admire athletic men, and no doubt the cult of athletics is encouraged thereby. Man is surely not going to adopt as his Queen of Beauty a sham Venus, with breasts only useful for giving a pleasing outline to her clothes.

When the fashion is so changed that a woman who cannot nurse her own baby is looked upon as a "poor thing" or a cripple, to be pitied, I fancy we shall hear less of this inability.

* See *The Nursling*, by Pierre Budin, translated by W. J. Maloney. (Caxton Publishing Co.)

